# Patterns and Outcomes of Acute Poisoning Among Children Presenting to Emergency Department in AlAhsa, Saudi Arabia

**DOI:** 10.7759/cureus.76565

**Published:** 2024-12-29

**Authors:** Hussain A AlGhadeer, Raghad Y Alturaifi, Jinan A AlGhafli, Mohammed H Alshawker, Alla M AlBisher, Fatimah A AlGhadeer, Alla A Bokhamseen, Ahmed Y AlGhadeer, Abdullah F Al Muaibid, Hassan A Alhajji, Ahmed A Al Shams, Salman F Aldubayyan, Abdullah A Alkhamees, Mohammed J Alsaeed, Fatimah A Alkhawajah

**Affiliations:** 1 Pediatrics, Maternity and Children Hospital, AlAhsa, SAU; 2 Pediatric Emergency Medicine, Maternity and Children Hospital, AlAhsa, SAU; 3 Pharmacology, King Faisal University, AlAhsa, SAU; 4 Pharmacology, National Guard Hospital, AlAhsa, SAU; 5 Pharmacology, King Fahad University Hospital, Khobar, SAU

**Keywords:** alahsa, chemical, children, drugs, household exposure, insecticides, intoxication, poisoning, saudi arabia

## Abstract

Introduction

Acute poisoning in children is still a global health concern that necessitates visiting the emergency department that might associated with morbidity and mortality. It has an impact on social, economic, and health issues, particularly for children under five who account for the majority of poisonings worldwide. Poisoning can result in mild cases, serious complications, or even death; oral ingestion is the most common way that poisoning occurs in children. This study will emphasize the necessity of putting such preventative measures into practice in order to lessen acute pediatric intoxication in the future. Furthermore, determining the important predictors of unintentional intoxication and the requirement for admission could help with the development of preventative strategies.

Aim

The present study is aimed to determine the pattern of poisoning and outcomes among the pediatric age group <14 years.

Methodology

A descriptive study based on retrospective records was carried out at the Maternity and Children Hospital in AlAhsa, Saudi Arabia, over a period of two years (August 2022 to August 2024), all cases of acute poisoning in children younger than 14 years old were included. Demographic details, the location, duration, type, and route of exposure to toxic agents, clinical presentation, course of treatment, and result are all included in the data analysis.

Results

In total, 158 cases were reported during the study period. The majority of acutely intoxicated children (136 (86%)) were aged one to <6 years. The highest incidence was observed between 12 a.m. and 6 a.m. (56 (35.4%)). Pharmaceutical ingestion (106 (67.1%)) caused more poisoning than chemical ingestion (52 (32.9%)). The most common drugs ingested were analgesics (28 (26.4%)) and cleaning agents were more among chemical poisons (25 (48.1%)). The majority of children (70.9%) were asymptomatic at the time of presentation. The most common symptoms reported by symptomatic children were gastrointestinal clinical manifestations. More than half of acute intoxication patients were discharged home, with 26 (16.5%) admitted to the general ward versus 22 (13.9%) admitted to the intensive care unit.

Conclusion

Physicians and public health authorities must conduct routine surveillance to update prevention and management strategies for pediatric poisoning. To reduce the frequency of pediatric intoxication, caregivers' knowledge of the potentially dangerous toxic agents and the risk factors for the condition should be improved. Additionally, efficient preventative measures should be put into place.

## Introduction

Acute poisoning is the term used for negative consequences that result from acute exposure to a toxic material, such as chemicals, pharmaceuticals, biological agents, environmental and occupational toxins, or due to drug usage by ingestion, inhalation, injection, or absorption through skin contact in amounts that are detrimental to the body [1,2]. Children are prone to poisoning at a high rate. Because drugs, detergents, and chemicals are easier for young children to access and because they are more likely to put objects in their mouths, the majority of studies' findings have shown that children under the age of five are more likely than older children to become poisoned [3-5]. The degree of pediatric poisoning and its consequences are impacted by a range of interconnected factors. These factors include the specific toxic substance involved, the amount consumed, the form of the toxin, the method of administration, the age of the child, exposure to one or multiple substances, and any concurrent medical conditions or injuries. Young children may experience accidental poisoning or be inadvertently exposed to toxins during medical treatment, while intentional poisoning is more common in adults [6].

The majority of pediatric poisonings involve non- or very mildly toxic substances, but on rare occasions, certain substances can be extremely toxic and need immediate, targeted medical attention to avoid serious injury or death. According to the most recent data available, the emergency department (ED) physician needs to be knowledgeable about managing poisoning and ready for the common causes of poisoning in children. Poisoning cases are often managed with decontamination, improved elimination, antidotes, and supportive care [7]. It is always a challenge for ED healthcare professionals to manage pediatric poisoning because the demographics and etiology can change over time, even within the same area. To identify trends in particular agents and other variables linked to childhood poisoning, regular surveillance is crucial [8].

Thus, acute poisoning is a significant global contributor to mortality and morbidity, with the incidence of mortality changing according to the cultural traits of various societies. Poisoning is a serious public health concern, despite being easily avoidable. Poisoning has a severe detrimental effect on a society's welfare in terms of both health and the economy [1,2,9]. As a result, determining risk factors is essential to lowering the frequency of poisoning and making sure the appropriate steps, such as prevention programs, are taken to improve patient outcomes.

Aim of the study

The goal of the current study is to identify the trends in poisoning and its effects on pediatric patients. Additionally, the study assessed the relationship between demographic factors and characteristics of the toxic agents.

## Materials and methods

Study design and population

A cross-sectional retrospective record-based descriptive study was conducted at a maternity and children hospital in AlAhsa, Saudi Arabia, over a period of two years (August 2022 to August 2024). The data were collected from the hospital’s electronic medical records. Children younger than 14 who visited the pediatric ED for poisoning were included. Patients more than 14 years old and children with doubtful poisoning where there was no clear etiology following a thorough evaluation by the principal investigator were excluded from the study.

Data collection

A semi-structured datasheet containing various dependent and independent variables, such as demographics (age and gender), poison characteristics (type, potential poison, route, time of poisoning, and arrival time at hospital), medical history (signs and symptoms), management information, and poisoning parameters were collected from the patient’s records.

Data analysis

The data were collected, reviewed, and then fed to Statistical Package for Social Sciences version 26 (Released 2019. IBM Corp., Armonk, NY). All statistical methods were two-tailed with an alpha level of 0.05 considering significance if the P-value is less than or equal to 0.05. Descriptive analysis for categorical data was done using frequencies and percentages for children's demographic data, medical data, and medication history. Also, children's poisoning patterns, circumstances, and types were tabulated with laboratory assessment and intervention. Clinical signs and symptoms with the clinical outcomes were graphed. Crosstabulation was used to assess the distribution of poisoning circumstances and patterns by children's age and gender using the Pearson Chi-Square test and exact probability test for small frequency distributions.

## Results

A total of 158 children presented to the ER by poisoning claims were included. Children's ages ranged from one month to 12 years with a mean age of 2.7 ± 1.8 and most of them (86.1%) were aged one to five years old. Exact 95 (60.1%) were males. Also, the vast majority of the children had no chronic health problem but four (2.5%) had asthma, four (2.5%) were autistic and ADHD was also reported. As for medications, four (2.5%) had anti-asthma, two (1.3%) were on Aripiprazole and 151 (95.6%) were not on any prior medications (Table 1).

**Table 1:** Personal characteristics of study children presented to emergency department with poisoning (n=158)

Personal data	No	Percent
Age in years		
< 1 year	10	6.3
1 to 5 years	136	86.1
> 5 years to 12 years	12	7.6
Mean ± SD	2.7 ± 1.8	
Gender		
Male	95	60.1
Female	63	39.9
Chronic disease		
Asthma	4	2.5
Autism	4	2.5
Attention-deficit/hyperactivity disorder (ADHD)	2	1.2
None	148	93.7
Regular medication		
Anti-asthma	4	2.5
Aripiprazole	2	1.3
Risperdal	1	0.6
None	151	95.6

Most of the cases (67.1%; 106) ingested drugs, with a single exposure (94.9%). The most reported drugs were analgesics and antipyretic (26.4%), antihistaminic (12.3%), antihypertensive (12.3%), and antidiabetics (9.4%). In the case of chemicals, the most poisons were cleansing substance (25 (48.1%)), perfume (nine (17.3%)), and insecticide (seven (13.5%)). Exact 56 (35.4%) were exposed during 12 am - 6 am, 52 (32.9%) during 12 pm - 6 pm, and 42 (26.6%) during 7 pm - 11 pm. Unintentional exposure was reported for 149 (94.3%) children but (five (3.2%)) had intentional exposure. The solid form was the most reported 83 (52.5%) for poisons and the liquid was reported among 68 (43%). The vast majority of the children had the poison orally (156; 98.7%). Most poisoning incidents were at home (139; 88%) (Table 2). All children were stable on arrival.

**Table 2:** Poisoning pattern, circumstances, and types among children presenting to the emergency department (n=158)

Poisoning data	No	Percent
Type of poisoning		
Chemical	52	32.9
Drug	106	67.1
Number of poisoning		
Single	149	94.9
Multiple	8	5.1
If drug, which group		
Analgesics & antipyretic	28	26.4
Antihistaminic	13	12.3
Antihypertensive	13	12.3
Antidiabetics	10	9.4
Vitamins	9	8.5
Iron	7	6.6
Others	7	6.6
Asthma medication	6	5.7
Anticoagulant	6	5.7
Antiemetic	6	5.7
Antiepileptic	5	4.7
Antibiotics	4	3.8
Hormonal	3	2.8
If chemical, which group		
Cleansing substance	25	48.1
Perfume	9	17.3
Insecticide	7	13.5
Nicotine	4	7.7
Rodenticide	4	7.7
Cosmetic	3	5.8
Time of exposure		
12 am - 6 am	56	35.4
7 am - 11 am	8	5.1
12 pm - 6 pm	52	32.9
7 pm - 11 pm	42	26.6
Circumstance of exposure		
Unintentional	149	94.3
Intentional	5	3.2
Unknown	4	2.5
Physical form of poisoning substance		
Cream	1	0.6
Liquid	68	43.0
Powder	6	3.8
Solid	83	52.5
Route of exposure		
Oral	156	98.7
Inhalation	1	0.6
Dermal	1	0.6
Place of incidence		
Home	139	88.0
Farm	19	12.0

**Figure 1:**
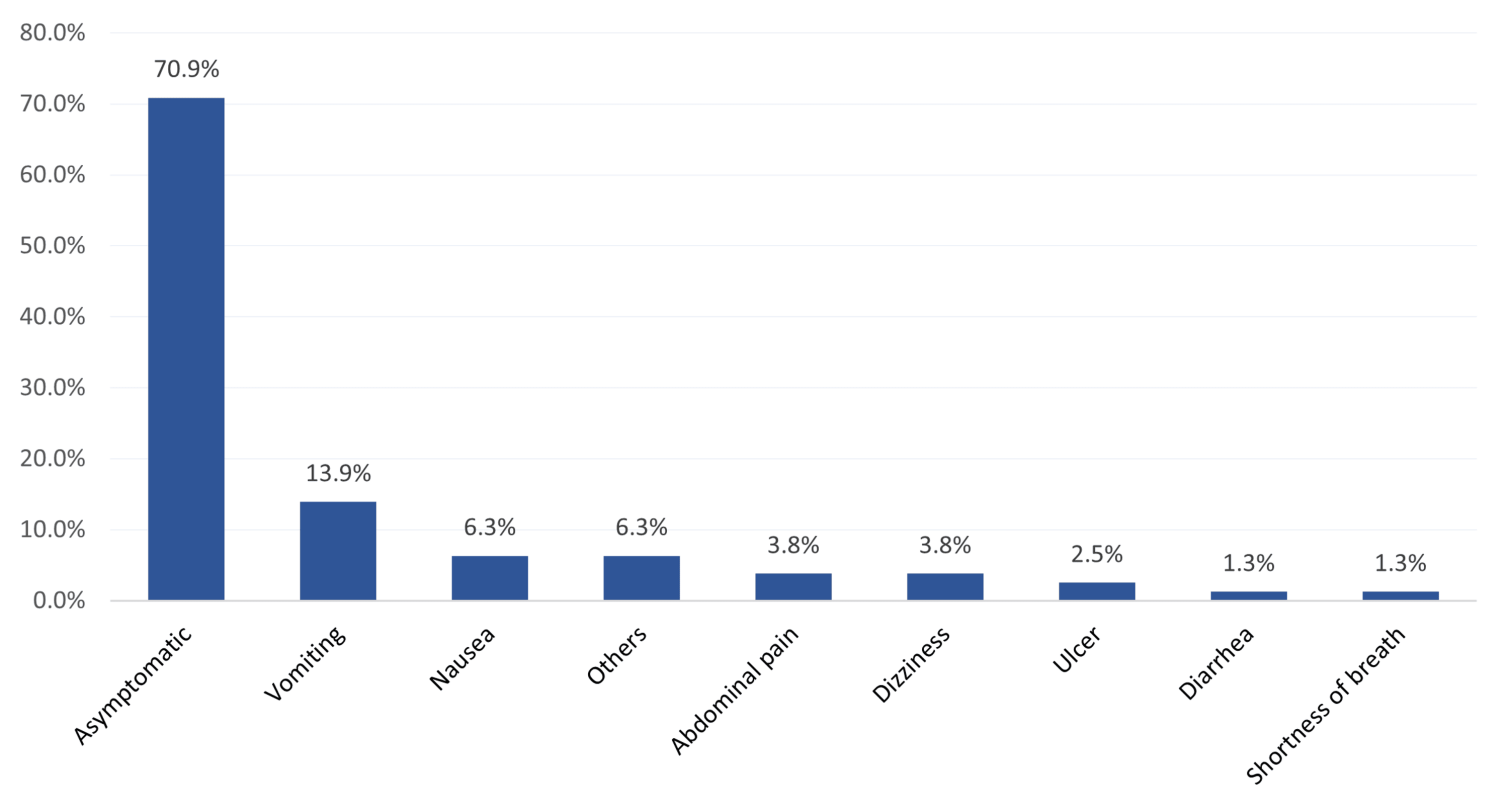
Clinical signs and symptoms among children presented to the emergency department with poisoning (n=158)

Considering clinical signs and symptoms among the study children, the most reported were vomiting (13.9%), nausea (6.3%), abdominal pain (3.8%), and dizziness (3.8%). Most of the children (70.9%) were asymptomatic (Figure 1).

Exact of 112 (70.9%) children underwent laboratory investigations mainly CBC (111 (99.1%)), Biochem (110 (98.2%)), venous blood gas (VBG) (110 (98.2%)), coagulation (32 (28.6%)), and ECG (30 (26.8%)). Most of the children showed normal laboratory findings (89.3%; 100). The most reported findings among positive cases were transaminitis (three (2.7%)), hypoglycemia (two (1.8%)), and acute kidney injury (two (1.8%)). A total of 51 (32.3%) children received activated charcoal as an antidote, but 102 (64.6%) did not receive any treatment or intervention. As for disposition, 102 (64.6%) cases were discharged to home, 26 (16.5%) were admitted to a general ward and 22 (13.9%) needed PICU (Table 3).

**Table 3:** Laboratory assessment, intervention, and management of children cases with poisoning CBC: complete blood count, ECG: Electrocardiography, VBG: venous blood gas

Laboratory findings	No	Percent
Laboratory investigation		
Yes	112	70.9
No	46	29.1
Laboratory investigation (n=112)		
CBC	111	99.1
Biochem	110	98.2
VBG	110	98.2
Coagulation	32	28.6
ECG	30	26.8
X-ray	28	25
Results of the investigation (n=112)		
Normal	100	89.3
Transaminitis	3	2.7
Hypoglycemia	2	1.8
Acute kidney injury	2	1.8
Acute liver failure	1	0.9
Hypokalemia, hyperglycemia	1	0.9
Metabolic acidosis, acute kidney injury	1	0.9
Metabolic acidosis, hypokalemia	1	0.9
Metabolic acidosis, transaminitis	1	0.9
Antidote used		
None	102	64.6
Activated charcoal	51	32.3
Activated charcoal with n-acytlesistien	4	2.5
Diphenhydramine	1	0.6
Deposition		
Discharge	103	64.6
Admission to general ward	26	16.5
Admission to intensive care unit	22	13.9
Discharge against medical advice (DAMA)	7	5.1

No significant difference was reported by age for most of the poisoning data except for circumstance of exposure and route of exposure where intentional exposure was reported for three children (25%) aged six to 12 years versus two children (1.5%) aged one to five years and none of the children aged < 1 year (P=0.001). Also, two children aged less than one year had inhaled or dermal poisons versus none of the others (P=0.001) (Table 4).

**Table 4:** Distribution of poisoning circumstances and pattern by children's age P: Exact probability test * P < 0.05 (significant)

Poisoning data	Age in years						P-value
	< 1 year		1-5 years		6-12 years		
	No	Percent	No	Percent	No	Percent	
Type of poisoning							0.167
Chemical	6	60.0	42	30.9	4	33.3	
Drug	4	40.0	94	69.1	8	66.7	
Number of poisoning							0.671
Single	10	100.0	128	94.8	11	91.7	
Multiple	0	0.0	7	5.2	1	8.3	
Time of exposure							0.197
12 am - 6 am	7	70.0	47	34.6	2	16.7	
7 am - 11 am	1	10.0	6	4.4	1	8.3	
12 pm - 6 pm	1	10.0	46	33.8	5	41.7	
7 pm - 11 pm	1	10.0	37	27.2	4	33.3	
Time from exposure to arrival to hospital							0.143
< 1 hour	4	40.0	63	46.3	2	16.7	
1-3 hours	4	40.0	57	41.9	5	41.7	
4-6 hours	1	10.0	10	7.4	3	25.0	
7-12 hours	0	0.0	1	0.7	0	0.0	
12-24 hours	1	10.0	1	0.7	1	8.3	
> 24 hours	0	0.0	4	2.9	1	8.3	
Circumstance of exposure							0.001*
Unintentional	9	90.0	133	97.8	7	58.3	
Intentional	0	0.0	2	1.5	3	25.0	
Unknown	1	10.0	1	0.7	2	16.7	
Route of exposure							0.001*
Oral	8	80.0	136	100.0	12	100.0	
Inhalation	1	10.0	0	0.0	0	0.0	
Dermal	1	10.0	0	0.0	0	0.0	

Only circumstance of exposure was significantly associated with children's gender as five male children (5.3%) had intentional poisoning compared to none of the females (p=0.049). gender using the Pearson chi-square test and exact probability test for small frequency distributions (Table 5).

**Table 5:** Distribution of poisoning circumstances and pattern by children's gender P: Exact probability test * P < 0.05 (significant)

Poisoning data	Gender				P-value
	Male		Female		
	No	Percent	No	Percent	
Type of poisoning					0.344
Chemical	34	35.8	18	28.6	
Drug	61	64.2	45	71.4	
Number of poisoning					0.559
Single	90	95.7	59	93.7	
Multiple	4	4.3	4	6.3	
Time of exposure					0.610
12 am - 6 am	32	33.7	24	38.1	
7 am - 11 am	4	4.2	4	6.3	
12 pm- 6 pm	35	36.8	17	27.0	
7 pm -11 pm	24	25.3	18	28.6	
Time from exposure to arrival to hospital					0.609
< 1 hour	42	44.2	27	42.9	
1-3 hours	39	41.1	27	42.9	
4-6 hours	8	8.4	6	9.5	
7-12 hours	0	0.0	1	1.6	
12-24 hours	3	3.2	0	0.0	
> 24 hours	3	3.2	2	3.2	
Circumstance of exposure					0.049*
Unintentional	88	92.6	61	96.8	
Intentional	5	5.3	0	0.0	
Unknown	2	2.1	2	3.2	
Route of exposure					0.511
Oral	93	97.9	63	100.0	
Inhalation	1	1.1	0	0.0	
Dermal	1	1.1	0	0.0	

## Discussion

The current study aimed to determine the pattern of poisoning and outcomes among pediatric age groups. Poisoning is defined as the exposure of an individual to substances that can cause symptoms and signs of organ dysfunction, potentially resulting in injury or death [10-12]. While this can be both accidental and non-accidental in younger children, it is more intentional in older children, particularly in high-income countries [13,14].

The current study showed that most child poisoning cases were males aged one to five years. Also, most of them were free of medical health problems with no medication history. Similar findings were reported by Areprekumor et al. [15] in Nigeria where more than half of the children were males and aged less than five years. Also, these results were consistent with many other studies in Sagamu, Umuahia, Warri, Ekiti, Enugu, Ghana, and South Africa [13,16-19]. According to an earlier study carried out in Sri Lanka, most children who consumed poisons were younger than five years old [20]. The WHO also reported an overall higher rate of poisoning in boys than girls in different world regions [21]. In Makkah, Saudi Arabia, Althobaiti et al. [22] found that 59% of the poisoning children's cases were males with a mean age of children was 5.2 years. Also, a 2018 Saudi study in Riyadh City found that 51.3% of children cases were males, with an average child age of 2.7 ± 2.1 years, consistent with the current study findings [23].

With regard to poisoning patterns, circumstances, and types, the study revealed that most poisons were drugs mainly analgesics and antipyretics that were taken for once and accidentally among most children. Cleansing substances were the chemicals most commonly taken, as most cases were at home. Time of poisoning was nearly uniformly distributed all over the daytime. The oral route was the main ingestion route for most cases with solid forms. In concordance with the current study, Alruwaili et al. [24] reported that child poisoning in Riyadh frequently involved a single agent of paracetamol in younger and older children. Similarly, Althobaiti et al. [22] reported that carbamazepine, methanol, risperidone, propranolol, and olanzapine as the main pharmaceutical products in child poisoning in Makkah. The most prevalent forms of poisoning were tablets occurred mostly through ingestion. In 2020, a Jeddah study discovered that the most common cause of acute poisoning was ingested medications (73.9%) [25]. This finding was in line with a prior study that indicated medicinal products were the primary source of poisoning [26]. Organophosphates and pesticides were also reported in many other studies [4,27,28]. The current study showed that poisoning was mainly accidental at home. This pattern can be ascribed to the possible poisoning agents stored in visually appealing, unsealed containers. This can pique the curiosity of small children, particularly when they are not receiving enough supervision. Similar results underlined the significance of safe storage procedures to avoid poisoning in children and were reported in many other studies [3,6,8,29].

The current study findings revealed that most cases were asymptomatic but very few cases had nausea, vomiting with abdominal pain, and dizziness. Laboratory findings were normal in most cases with antidotes (mainly activated charcoal) given to one-third of the children. These all are consistent with reported clinical findings and case stability among children [23-25]. In Alruwaili et al.'s study [24], two children (0.19%) died due to exposure to an organophosphorus compound resulting in cardiopulmonary arrest and death. Other studies in Saudi Arabia reported higher reported mortality rates ranging from 1.5% to 4.6% [23,24].

Limitation

Because of the retrospective collection method, some data may be missing. In addition, emergency physicians may have started to manage more poison cases without documentation. The study does not explore the socio-economic or educational background of caregivers, which could provide additional insights into risk factors. It lacks data on the accessibility and availability of toxic substances in the household environment. Despite these limitations, our study was based on results that emphasize the importance of developing a national surveillance strategy for monitoring and managing poisoning events in Saudi Arabia.

## Conclusions

The current study showed that child poisoning was most frequent among males under the age of five years (age of exploration) mainly due to incidental ingested drugs. Most children were stable and symptomatic received antidotes and were discharged to home with no complications. Adopting a national policy that ensures timely and appropriate management will yield positive results. Furthermore, families in particular need to pay greater attention to educating their members about the importance of following safety precautions at home in light of children's exposure to dangerous chemicals. Future research is required to elucidate the significance of the different childhood-related factors.
